# Meta-Alignment with Crumble and Prune: Partitioning very large alignment problems for performance and parallelization

**DOI:** 10.1186/1471-2105-12-144

**Published:** 2011-05-10

**Authors:** Krishna M Roskin, Benedict Paten, David Haussler

**Affiliations:** 1 Department of Computer Science, Univ. of California, Santa Cruz, USA; 2 Center for Biomolecular Science & Engineering, Univ. of California, Santa Cruz, USA; 3 Howard Hughes Medical Institute, Univ. of California, Santa Cruz, USA

## Abstract

**Background:**

Continuing research into the global multiple sequence alignment problem has resulted in more sophisticated and principled alignment methods. Unfortunately these new algorithms often require large amounts of time and memory to run, making it nearly impossible to run these algorithms on large datasets. As a solution, we present two general methods, Crumble and Prune, for breaking a phylogenetic alignment problem into smaller, more tractable sub-problems. We call Crumble and Prune *meta-alignment *methods because they use existing alignment algorithms and can be used with many current alignment programs. Crumble breaks long alignment problems into shorter sub-problems. Prune divides the phylogenetic tree into a collection of smaller trees to reduce the number of sequences in each alignment problem. These methods are orthogonal: they can be applied together to provide better scaling in terms of sequence length and in sequence depth. Both methods partition the problem such that many of the sub-problems can be solved independently. The results are then combined to form a solution to the full alignment problem.

**Results:**

Crumble and Prune each provide a significant performance improvement with little loss of accuracy. In some cases, a gain in accuracy was observed. Crumble and Prune were tested on real and simulated data. Furthermore, we have implemented a system called Job-tree that allows hierarchical sub-problems to be solved in parallel on a compute cluster, significantly shortening the run-time.

**Conclusions:**

These methods enabled us to solve gigabase alignment problems. These methods could enable a new generation of biologically realistic alignment algorithms to be applied to real world, large scale alignment problems.

## Background

Multiple sequence alignment methods are a major tool in comparative genomics. The alignments they generate are primary data for a wide array of analyses: discovery of evolutionarily conserved elements [1,2], identification of functional RNAs [3], reconstruction of evolutionary events [4] to name a few.

New high throughput sequencing methods are greatly increasing the amount of available sequence information. To take full advantage of this wealth of data, current multiple sequence alignment methods will need to be adapted to handle larger datasets.

We present two general methods to adapt current global alignment algorithms to large scale problems. This will enable current methods to be used for larger and larger problems and also allow computationally expensive methods to be applied to biologically relevant problems.

## Related Work

Segmentation methods have been used to align full genomes. To align the mouse genome to the human genome, Schwartz et al. divided the human genome into approximately 3,000 segments of ~ 1.01 Mb with a 10 kb overlap between adjacent segments [5]. They hypothesize that any alignment that extends for 10 kb is almost certain to contain an alignment that would bridge the adjacent segments and thus no alignment will be lost by the segmentation. Crumble is a more principled segmentation method that, given some assumptions, guarantees that each segment contains all the sequence necessary for correct alignment.

Dress et al. describe a divide and conquer method that recursively subdivides three sequence alignment problems length-wise to produce shorter alignment problems [6]. They outline how their method might be extended to more sequences but since it is based on considering all pairs of sequences, it is not applicable to beyond moderately sized problems. Reinert et al. use the above method in an iterative framework that allows the an alignment to be refined until the alignment score stops improving or time runs out [7]. This methods is also too computationally expensive to apply to large datasets.

The MISHIMA algorithm of Kryukov and Saitou starts by finding a non-conflicting set of *k*-mers shared by all sequences [8]. The sequences between these *k*-mers are then aligned independently and concatenated to form the complete alignment. Crumble uses a more general method that does not require the constraints to span all sequences. Thus Crumble can be used to align more diverged sequences where it is impossible to find *k*-mers shared by all sequences. Crumble also takes advantage of parallelization while MISHIMA currently does not.

The method employed by Prune can be considered a generalization of the progressive alignment methodology [9,10]. Progressive alignment proceeds by merging sibling nodes to form the alignment of the parent. Thus it only moves up the tree one node at a time. Prune, on the other hand, can move up the tree several nodes at a time. Progressive alignment also uses leaf sequences to guide the merging process while Prune uses a more general strategy.

Several authors have extended specific alignment algorithms to take advantage of parallelization, usually thread or intra-core parallelization [11-13]. These methods are not designed to take advantage of cluster level parallelization. Furthermore they are specific to a given alignment program. The open framework we present here is designed to leverage parallelization for a general class of multiple alignment programs. Thus it can be used to enable the parallelization of many current and future global alignment algorithms.

## Implementation

We adopt the general approach of dividing a large alignment problem into smaller sub-problems. The sub-problems are solved and the alignments are recombined to form a solution to the whole, original problem. We perform this division in such a way that most sub-problems are independent and thus can be solved in parallel. This can lead to reduced run-time because of smaller individual problems and because sub-problems can be solved in parallel on a multi-core machine or cluster. Smaller problems can also result in less memory consumption; allowing some methods to be applied at all.

An alignment problem can be large in two ways: large in sequence length or large in sequence number. *Crumble *deals with problems that are large in sequence length. It breaks up long alignment problems into shorter problems. *Prune *handles problems with a large number of sequences. It cuts up deep alignment problems into sub-problems with fewer sequences.

Our methods do not perform the actual alignment of sub-problems. Other programs are used for that part of the process. Almost any current global alignment algorithm can be adapted to work with Crumble and Prune by writing a simple wrapper. Thus we refer to Crumble and Prune as *meta-alignment *methods. In theory, both these methods would result in performance gains at the loss of some alignment accuracy. However, we found that very significant performance gains can be made with negligible loss of alignment accuracy and in a few cases some accuracy can be gained.

### Crumble: breaking long alignment problems into shorter sub-problems

**Figure 1:**
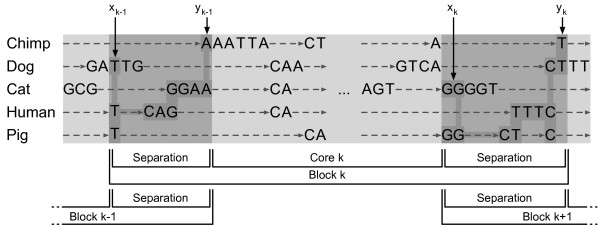
**A set of constraints visualized as a sparse alignment**. In each species, positions increase from left to right with respect to the partial order. Sequence positions in the same column are equal under the partial order. A set of *x_k_*, *y_k _*separations breaks the sparse alignment into a set of blocks. Positions *x*_*k*-1_, *y*_*k*-1 _and *x_k_*, *y_k _*define block *k*, which is composed of the core *k *(light gray) and the adjacent separation (dark gray).

Given a set of sequences, we begin by generating a system of sequence constraints. These constraints align a base position in one sequence with a base position in another sequence [14]. Constraints of this form can be thought of as a sparse alignment and have been used in several alignment programs [15-17]. The constraints impose a partial ordering ≺ on the sequences [18]. Under this partial ordering, aligned positions are equal and base positions along the same sequence are increasing with respect to the partial ordering. Given a base position *x *in a sequence, Crumble searches for a set of base positions *A *= {*a*_1_, *a*_2_, ..., *a_n_*} in each of the *n *species being aligned such that *x *≼ *a_i _*for all *i *(Figure 1). We now consider the constraints as a sparse alignment and let *y *be the right-most position in *A*, i.e. *y *such that *a_i _*≼ *y *for all *i*. The sequences between *x *and *y*, inclusive, forms a *separation *that divides the alignment into three classes: sequences to the left of *x*; *x*, *y*, and the sequences between them; and sequences to the right of *y*. No base position less that *x *in the partial ordering may be aligned with a base position greater than *y*. Such an alignment would contradict the partial ordering imposed by the constraints. Note that if *x *= *y*, then the separation is simply a column of fully aligned sequence in all species.

Crumble tiles the constraints with a set of positions

where each pair *x_i_*, *y_i _*forms a separation as described above. The sequences between *y*_*k*-1 _and *x_k _*compose a *core*. As noted above, each core is independent in the sense that sequences in one core cannot be aligned with sequences in another core. The position of the sequences between cores is ambiguous. Both sets of adjacent sequences need to be considered when aligning a core. Thus we define a *block *as a core together with its flanking separations (Figure 1, bottom). The pairs *x_i_*, *y_i _*are chosen to maintain a user-selected approximate core size. The exact size of a block will depend on the set of initial constraints. To prevent blocks from becoming too large, a user-defined maximum block size can also be set. If the maximum is reached, the block is truncated to the maximum block size. The truncated sequence becomes part of the next block.

**Figure 2:**
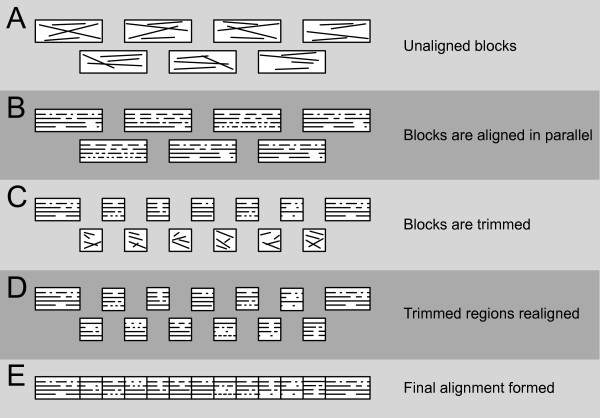
**The Crumble pipeline**. The pipeline used after the formation of semi-independent blocks (A). Blocks are aligned (B) and trimmed to remove overlap (C). Overlaps are aligned (D), and the final alignment is formed by concatenation (E). Note that the alignments in (B) and (D) can be performed in parallel.

Because the blocks are semi-independent, an alignment of all sequences is constructed as follows: each block is aligned in parallel using a user-specified global alignment method; each aligned block is then trimmed until there is no sequence overlap between adjacent blocks; then the trimmed off sequences are realigned; and, finally, all sub-alignments are concatenated to form the full alignment (Figure 2).

The method used to generate the sparse constraints is user-definable. By writing a simple wrapper, any current alignment constraint method can be used. If the constraints are a subset of the true alignment, then the blocks produced by Crumble contain all the sequence necessary for their correct alignment.

### Prune: trimming deep alignment problems into smaller sub-problems

**Figure 3:**
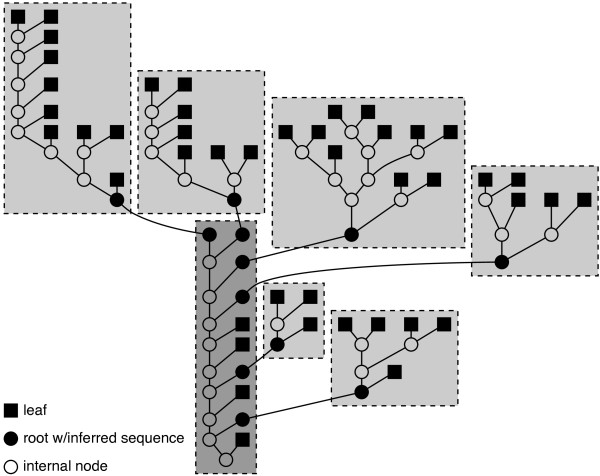
**Prune partitioning of a phylogenetic tree of 44 species**. Prune run with a maximum sub-tree size of 10 sequences breaks the tree into seven sub-trees. Six of the sub-trees (light gray) can be aligned in parallel because they contain only known leaf sequences (filled squares; out-groups for sub-trees are not shown). Once these six sub-trees are aligned and the sequence of the roots (filled circles) is inferred, the internal sub-tree (dark gray) can be aligned. Note that this sub-tree includes both leaf sequences and inferred sequences. The alignments from the light gray sub-trees are merged with the alignment of the dark gray sub-tree to form the alignment of the entire tree.

Given a set of sequences and a rooted phylogenetic tree, Prune breaks the tree into sub-trees that overlap at an internal node. Prune infers a sequence for the root of each sub-tree, i.e., the overlapping nodes. Using inferred sequence at an internal node breaks the conditional dependencies between the sub-trees. In a leaf-to-root fashion, Prune aligns the sequences in each sub-tree as well as the sequences of any inferred nodes included in the sub-tree. A sequence is then inferred for the root of the sub-tree. This inferred sequence is used in the alignment of the sequences in the parent's sub-tree. Once all sub-tree alignments have been formed, the alignments of each sub-tree are merged using the inferred overlapping root sequence as a guide (Figure 3). To provide information about the rest of the tree, an out-group sequence from a leaf node closest to the sub-tree root, as measured by branch length, is included when aligning each sub-tree. Prune forms the sub-trees so as to minimize the number of stages that must be performed in sequence while enforcing a maximum number of sequences in each sub-tree, including inferred sequences. If the tree nodes are numbered 1, 2, ..., *N *and *R *is the root node, then the minimum number of stages needed with a maximum of *M *sequences per sub-tree is given by *S*_*R*1_. *S_ij _*is defined by the following recurrence relation:

where *r_i _*and *l_i _*are the right and left children of internal node *i *respectively. Informally, *S_ij _*is the minimum number of stages needed if the sub-tree rooted at node *i *has *j *sequences (leaf or inferred) in it. The only sub-trees rooted at a leaf that make sense are those containing one node. Thus Equation (1) assigns all sub-trees rooted at leaf *i *containing only one sequence a stage count of 1. This trivial "alignment" of a single sequence is counted as a stage since time is spent extracting and processing the sequence. Equation (2) assigns all other trees below leaf *i *(but not rooted at *i*) a stage count of ∞. Equation (3) calculates the minimum number of stages needed if node *i *is the root of a sub-tree. It does this by considering all ways to merge the right (*r_i_*) and left (*l_i_*) children of node *i *into a new sub-tree while respecting the maximum number of sequences per sub-tree, *M*. It selects the merge that minimizes the number of stages at *i*. The new stage depth becomes one more than this minimum. Equation (4) calculates the minimum number of stages needed if the sub-tree below node *i *(but not rooted at *i*) contains exactly *j *sequences, leaf or inferred. Thus, *S*_*R*1 _is the minimum number of stages needed for the entire tree if the global root node *R *is the root of a sub-tree. The above recurrence relation can be calculated using a dynamic programming algorithm and the sub-trees that achieve the minimum number of stages can be inferred. For a given maximum number of sequences per sub-tree, the dynamic programming calculation takes time linear in the number of species.

**Figure 4:**
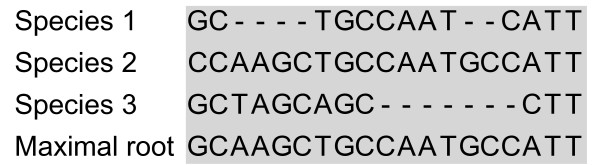
**Example of the Maximal root inference method**. Every alignment column is assigned the most frequently occurring base in the column. Thus Maximal infers the longest possible root sequence that fits within the alignment.

Originally, two methods were used to infer the root sequence: Ortheus [19] and *Maximal*, a heuristic that assumed that the most commonly occurring base in an alignment column was the base for that position in the root. *Maximal *is such named because it infers the longest possible root sequence that fits within the alignment (Figure 4). Surprisingly, we found that Maximal performed better that Ortheus for this particular application (Table 1). It is possible that the Maximal method, while less biologically meaningful, provides more opportunities for homologous positions to be aligned. Therefore, we adopted Maximal as the default inference method and performed our benchmarking using it.

**Table 1:** Comparison between root inference methods.

		50 leaves Agreement	
		
	Number Nodes	Maximal	Ortheus
Prune w/Pecan	30	0.880	0.579
	15	0.909	0.560
	7	0.912	0.555

Prune w/FSA	30	0.912	0.574
	15	0.893	0.523
	7	0.885	0.495

Prune w/MUSCLE	30	0.899	0.579
	15	0.896	0.555
	7	0.905	0.501

The average agreement score of Prune alignments when Maximal and Ortheus root inference methods are used. Fifty alignment problems with fifty leaf species and ~10 kilobases of sequence were used. Three underlying alignment algorithms and three different maximum sub-tree sizes were used in the comparison. The faster Maximal method performed better across all comparisons that Ortheus for this application.

The sub-tree alignment method and the inference method are user-definable. The maximum number of sequences in each sub-tree is also user-configurable.

As discussed above, Prune can be considered a generalization of progressive alignment. The two are equivalent if, in Prune, the maximum number of nodes per sub-tree is set at two, the inference method selects only leaf sequences, the addition of out-groups is disabled, and parallelization is disabled.

### Job-tree: Solving gigabase alignment problems with Prune and Crumble

Prune and Crumble can be used together to align long and deep alignment problems. To take advantage of both Prune and Crumble's parallelization, a cluster system with the following functionality is needed: any job must be able to spawn its own set of parallel jobs. This is because Prune's sub-tree alignment tasks are run in parallel and each of those tasks executes Crumble which in turn spawns its own set of parallel jobs. This requires a hierarchical and dynamic job system.

**Figure 5:**
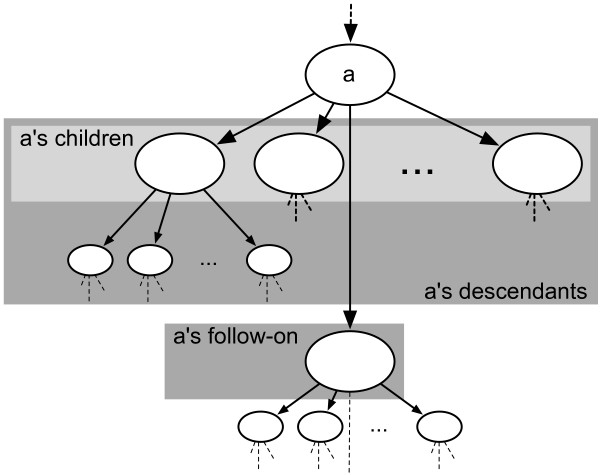
**Schema of the Job-tree job system**. In Job-tree, job *a *creates a set of jobs that perform a task in parallel. These jobs are collectively called the *children *of job *a*. The job also creates a *follow-on *job to be performed after all children have successfully completed. The follow-on job is responsible for cleaning up the input files created for the children and for any further processing. After job *a *ends successfully, the batch system runs the children. These jobs may, in turn, have children and follow-on jobs. Upon completion of all descendants, the follow-on job is run. The follow-on job may create more children.

Job-tree is a batch system designed to manage jobs in a cluster running on top of an existing batch system, such as Parasol [20], LSF [21], or Sun Grid Engine [22]. Job-tree makes it simple for jobs to dynamically create new jobs in a hierarchical fashion (Figure 5).

Communication between Job-tree and the jobs proceeds via XML files that are processed before and after each job. Job-tree is currently implemented in Python and works on top of the Parasol batch system but can be extended to work with other job systems. It also features a serial mode that runs on a single machine, which is useful for testing or for small tasks.

## Results and Discussion

We used two methods to measure the effect that Crumble and Prune had on alignment accuracy and run-time. The first method uses a large collection of alignment problems generated by simulation. Alignment problems generated by simulation provide "true alignments" that can be used to measure the quality of predicted alignments. We created a large collection of simulated datasets to test Crumble and Prune. The second method uses real biological data. We tested Crumble by aligning sequence from six species to a 90 kb block of the human genome. Prune was tested on twelve alignment problems from the Rfam database that contain more than 200 sequences [23]. Datasets with fewer sequences can be easily and quickly aligned by current methods and thus are not on the scale of problem that Prune was designed to address.

### Simulation Results

All computations in this section were performed on an AMD based cluster. Each compute node had two AMD Opteron 246 HE processors running at 2 GHz with 4GB of memory. The largest number of parallelizable jobs was 26. While hard to guarantee in a shared work environment, there were sufficient nodes available to allow most parallelizable jobs to be run in parallel.

#### Crumble

We used a published dataset of simulated sequences [24]. The dataset contained 50 alignment problems over nine species with ~60 kilobase of sequence per species. These alignments were generated using a model of neutral evolution and have been used to measure the alignment accuracy of several other methods [24,17,25,16]. Using the same simulation program, we generated additional longer datasets with ~150 kilobases, ~500 kilobases, and ~1 megabase of sequence over the same nine species tree as used in previous studies. The simulation program was independently developed and models substitutions using the HKY model [26], deletions and insertions along each branch as well as retrotransposon insertions. For more details see the supplemental materials of Blanchette et al. [24]. The total length of the tree was 1.13 substitutions per site. Fifty simulations were run for each sequence size. In total, 200 alignment problems, totaling ~760 megabases of sequences, were used to test Crumble.

To measure the degree of similarity between predicted and true alignments, we used the *average agreement *score [24]. For pairwise alignments, the average agreement score (agreement for short) is as defined the fraction of positions of the predicted alignment that agree with the true alignment. There is agreement if the *i*-th nucleotide of species *X *is aligned to the *j*-th nucleotide of species *Y *in both the predicted and true alignment or if the positions are similarly aligned to gap characters. Thus, two identical alignments obtain an agreement score of 1, whereas two completely different alignments get a score of zero. For more than two species, the pairwise score is averaged over all pairs of species.

We calculated the agreement score resulting from Crumble breaking up the alignment problem into several fractional sized pieces (Table 2). We used Pecan [17], FSA [25], and MUSCLE [27] as the underlying alignment algorithms. The set of sparse constraints used to break up the alignment problem was generated with PrePecan, the constraint generation system employed by Pecan. As the block size decreased, Crumble broke the alignment problem into smaller and smaller sub-problems that were aligned in parallel using the Parasol job system. As the sub-problems decreased in size, the run-time decreased, as expected, with only a small loss in accuracy. For longer alignment problems, a much more pronounced performance gain was observed and, at the same time, the alignment accuracy was maintained. Some methods were unable to solve long alignment problems directly because of high memory usage. Crumble allows these methods to be applied to longer problems than were previously possible.

**Table 2:** Crumble results for different sized simulated datasets and underlying alignment methods.

		60 kb		150 kb		500 kb		1000 kb	
					
		Time	Agreement	Time	Agreement	Time	Agreement	Time	Agreement
Pecan^1^	3.43	0.896	10.6	0.905	46.9	0.906	100	0.906	
Crumble w/Pecan	60%	3.29	0.894	7.18	0.904	21.5	0.905	51.9	0.906
	30%	2.56	0.889	4.66	0.903	11.9	0.905	23.5	0.905
	15%	2.39	0.859	3.77	0.893	8.29	0.903	13.9	0.905

FSA^2^		37.4	0.886	_*a*	_*a*	_*a*	_*a*	_*a*	_*a*
Crumble w/FSA	60%	25.8	0.881	69.8	0.903	_*a*	_*a*	_*a*	_*a*
	30%	21.0	0.873	3act9.2	0.898	_*a*	_*a*	_*a*	_*a*
	15%	17.7	0.849	25.5	0.893	104.	0.811	_*a*	_*a*

MUSCLE^3^	_*a*	_*a*	_*a*	_*a*	_*a*	_*a*	_*a*	_*a*	
Crumble w/MUSCLE	60%	_*a*	_*a*	_*a*	_*a*	_*a*	_*a*	_*a*	_*a*
	30%	128	0.707	_*a*	_*a*	_*a*	_*a*	_*a*	_*a*
	15%	63.1	0.679	251.	0.705	_*a*	_*a*	_*a*	_*a*

^1 ^Pecan was run with default parameters.

^2 ^FSA was run with the --exonerate, --anchored, and --softmasked flags.

^3 ^MUSCLE was run with default parameters.

^*a *^The majority of these problems were unable to be aligned due to running out of memory.

The run-time and average agreement score of Crumble alignments of different sized datasets. Several sets of simulated alignment problems were generated using a root sequence of 60, 150, 500, and 1000 kilobases. The neutral evolution of each root sequence was simulated over a nine species tree. Fifty problems were generated per root size for a total of two hundred test alignment problems. The agreement and run-time (in minutes) for each problem size is the average over the fifty simulated alignments. Crumble was used to break the problems down to sub-problems that were 60%, 30%, and 15% of the length of the original problem. The approximate core size was set to 60%, 30%, and 15% of the length of the original problem and the block was allowed to be at most 4 kb larger as measured in any of the sequences. Pecan, FSA, and MUSCLE were used as the underlying alignment method. PrePecan was used to generate the constraints. We were unable to apply FSA directly (not using Crumble) to 150 kb or larger problems because FSA required more than the 4GBs of memory we had available per cluster node. Using Crumble we were able to run FSA on problems as large as half a megabase. MUSCLE had more memory issues but we were able to use it on problems as large as 150 kb using Crumble. For Pecan, Crumble achieved more than a seven fold speedup with almost no loss of accuracy on the largest problem size.

#### Prune

A similar methodology was used to evaluate the alignment accuracy achieved using Prune. The neutral evolution of ~10 kilobase of DNA on a 50, 100, 500, and 1000 species tree was generated using the same simulation program used above. Trees were generated with Bio::Tree::RandomFactory module of BioPerl [28]. Lacking data on retrotransposon insertions into large clades, we disabled the retrotransposon modeling in the simulation program. To compensate for the lack of complexity, we greatly increased the tree length to 187.8, 368.8, 709.2, and 925.9 substitutions per site for the 50, 100, 500, and 1000 leaf trees respectively. Pecan, FSA and MUSCLE were employed for the underlying alignment algorithm. We also compare Prune to MAFFT [29] and SATé [30] which specialize in many species multiple alignment problems.

Unlike for Crumble, we do not expect to see a monotonic decrease in running time as Prune breaks the tree into smaller and smaller sub-trees. While smaller sub-trees will results in faster alignment of the sub-problems, it can also result in more alignment stages. This is different from Crumble because the number of stages in Crumble is fixed. We have observed this non-monotonicity of running times (Table 3). Unexpectedly, we also see non-monotonicity in agreement as Prune breaks the tree into smaller and smaller sub-trees. In some cases, better agreement is actually achieved with smaller sub-trees. We hypothesize that, on deep alignments, some methods discard large amounts of information in order to fit the problem in memory. On smaller sub-trees more information can be retained which results in an increase agreement that outweighs any loss that comes from considering only a sub-tree. In general, as the sub-trees decreased in size, the run-time decreased significantly with only a small loss in accuracy.

**Table 3:** Prune results for different sized datasets and underlying alignment methods.

		50 leaves		100 leaves		500 leaves		1000 leaves	
					
		Time	Agreement	Time	Agreement	Time	Agreement	Time	Agreement
Pecan^1^		21.9	0.914	297.	0.879	_*a*	_*a*	_*a*	_*a*
Prune w/Pecan	60%	7.26	0.880	39.2	0.862	_*a*	_*a*	_*a*	_*a*
	30%	3.13	0.909	19.6	0.839	_*a*	_*a*	_*a*	_*a*
	15%	7.26	0.912	13.3	0.878	125.	0.844	_*a*	_*a*
	7%	4.24	0.909	13.5	0.849	29.1	0.907	122.	0.877

FSA^2^		63.1	0.933	266.	0.856	_*a*	_*a*	_*a*	_*a*
Prune w/FSA	60%	33.8	0.912	78.9	0.838	589.	0.871	_*a*	_*a*
	30%	10.5	0.893	23.8	0.838	142.	0.879	_*a*	_*a*
	15%	4.25	0.885	17.1	0.857	40.8	0.877	150.	0.861
	7%	3.00	0.866	4.23	0.842	12.7	0.903	34.8	0.887

MUSCLE^3^		55.6	0.905	138.	0.799	_*b*	_*b*	_*b*	_*b*
Prune w/MUSCLE	60%	40.7	0.899	77.9	0.777	886.	0.862	_*b*	_*b*
	30%	24.7	0.896	42.8	0.777	368.	0.883	_*b*	_*b*
	15%	15.1	0.905	29.1	0.828	185.	0.899	440.	0.900
	7%	24.7	0.905	18.8	0.841	114.	0.924	228	0.928

MAFFT^4^		3.17	0.897	5.39	0.806	20.1	0.886	25.2	0.912

SATé^5^		101.	0.915	301.	0.840	_*b*	_*b*	_*b*	_*b*

^1 ^Pecan was run with default parameters.

^2 ^FSA was run with the --exonerate, --anchored, --softmasked, and --fast flags.

^3 ^MUSCLE was run with default parameters.

^4 ^MAFFT was run with the --treein option.

^5 ^SATé was run with the -t option but limited to two iterations. We found that more iterations did almost nothing for accuracy.

^*a *^The majority of these problems were unable to be aligned due to running out of memory.

^*b *^The majority of these problems took longer than 3 days and were aborted.

The run-time and average agreement score of Prune alignments of different sized datasets. Several sets of simulated alignment problems were generated using a root sequence of 10 kilobases. The neutral evolution of each root sequence was simulated over 50, 100, 500, and 1000 species trees. Fifty problems were generated per tree size for a total of two hundred test alignment problems. The agreement and run-time (in minutes) for each problem size is the average over the fifty simulated alignments. Each underlying alignment method was tested on the dataset (Pecan, FSA, MUSCLE). Prune was then used to break the problems down into sub-trees that contained at most 60%, 30%, 15%, and 7% of the nodes in the entire tree. The largest number of stages was six but most of the problems had no more than 3 stages. Pecan, FSA, and MUSCLE were used as the underlying alignment method to Prune. We also performed alignment using MAFFT and SATé to compare against. To ensure a fair comparison, the true tree topology was passed to SATé (using -t option) and to MAFFT (using the poorly documented --treein option). We were unable to apply some alignment algorithms to large problems because of very long run-times and memory issues. Using Prune, we were able to use Pecan, FSA, and MUSCLE to solve alignment problems that were much deeper than could be solved without Prune. Prune achieved a very large speedup with little loss of accuracy and sometimes with an increase in accuracy.

#### Job-tree

Using the Job-tree system, we were able to apply Pecan to gigabase size alignment problems. The neutral evolution of a one megabase root sequence was simulated on a 1000 species tree. Prune was used to break the tree into sub-trees no larger than 10 nodes. Crumble was then used to break each 10 species sub-problem into 100 kilobase chucks that were then aligned with Pecan. All the sub-problems were then assembled to form a solution to the entire gigabase alignment problem.

Each of the fifty gigabase alignment problems took just over nine hours to solve. No other tested alignment method was able to align problems of this magnitude. To calculate the average agreement score, we were forced to randomly sample the pairs of sequences instead of averaging over all  pairs. Using this method, the agreement score for these solutions was calculated as 0.754. Since no other methods were able to solve an alignment problem of this scale, we have nothing with which to compare.

### Biological Data

The above simulation studies provide a comprehensive look at the effect Crumble and Prune have on performance. It is also enlightening to look at performance on "real world" datasets.

The main problem with real world alignment problems is evaluation. Unlike simulated data, there is no clear alignment with which to compare. For RNA and protein families, there does exist large, commonly used databases of multiple alignment problems that are used for benchmarking. The reference multiple alignments in these databases are usually hand curated multiple alignments generated by an ensemble of alignment tools. These reference multiple alignments are commonly used as the "true alignment" and used to measure the quality of predicted alignments. We used alignment problems from the Rfam database of RNA multiple alignments to test Prune on real, biological data [23].

For genomic scale problems, there is currently no large, commonly used database of genomic scale multiple alignment problems. The sequences in Rfam are very short (<1.2 kb) and thus not useful for testing Crumble. Thus, we used a different method to evaluate Crumble on long alignment problems. We extracted and aligned a 90 kb region of the human genome and the orthologous region from six other species. To get an idea of alignment accuracy, we calculated the log-likelihood of the alignment. We used phyloFit from the PHAST package to calculate the maximum log-likelihood of the alignment [31]. While the tree topology was fixed, phyloFit was allowed to vary branch lengths when calculating the maximum log-likelihood of each alignment. We used the REV base substitution model where gaps are treated as missing data [32]. While log-likelihood values closer to zero only represent higher likelihood that the alignment was generated by the given phylogenetic model, we believe the log-likelihood is a reasonable, if ad hoc, method of assessing alignment quality when no good reference alignment is available.

The computations in this section were performed on a different cluster than the one used for the results of Section. That cluster has been reappropriated. The cluster used for this section has eight nodes each with two dual-core AMD Opteron 2214 HE running at 2.2 GHz and 32GB of memory.

#### Crumble

We examined Crumble's effect on performance by aligning genomic DNA from 7 species. Looking at the UCSC Genome browser, we selected a ~90 kb region on the human genome, chr14:104721193-104812803 [33]. This region was selected because it contained some but not an excessive number of rearrangements. Thus it was a reasonable problem for global alignment algorithms. Using the "Chain/Net" tracks [34], we found the best matching region in cow (bosTau4 assembly), dog (canFam2), mouse (mm9), chimp (panTro2), macaque (rheMac2), and rat (rn4) and extracted the corresponding sequence to form the alignment problem.

As above, Crumble was used to break the alignment problem into several fractional sized pieces (Table 4) and Pecan [17], FSA [25], and MUSCLE [27] were used as the underlying alignment algorithms. The set of sparse constraints was generated with PrePecan. The log-likelihood of the alignment was used to measure alignment quality. As on the simulated data, Pecan running by itself produced the best alignments under the log-likelihood measure. Crumble running on top of Pecan was able to halve the running time with a very small loss in log-likelihood. Crumble running with FSA achieved greater performance gains over FSA with even less decrease in the log-likelihood score. MUSCLE was unable to solve this alignment problem without the aid of Crumble.

**Table 4:** Crumble results for 90 kb of genomic DNA from seven species.

		Time	Log-likelihood^1^
		
Pecan^2^		11.3	-0.354
Crumble w/Pecan	60%	7.42	-0.355
	30%	4.67	-0.357
	15%	5.42	-0.357
FSA^3^		38.3	-0.374
Crumble w/FSA	60%	20.4	-0.375
	30%	12.2	-0.375
	15%	9.68	-0.376

MUSCLE^4^		_*a*	_*a*
Crumble w/MUSCLE	60%	_*a*	_*a*
	30%	153.	-0.363
	15%	59.2	-0.367

^1 ^The log-likelihood of the alignment as calculated by phyloFit, in millions of nats.

^2 ^Pecan was run with default parameters.

^3 ^FSA was run with the --exonerate, --anchored, --softmasked, and --fast flags.

^4 ^MUSCLE was run with default parameters.

^5 ^This problem was unable to be aligned due to running out of memory.

The run-time and log-likelihood score of Crumble alignments. Each underlying alignment method (Pecan, FSA, MUSCLE) was tested on the dataset. Crumble was then used to break the problem into sub-problems that were approximately 60%, 30%, and 15% of the length of the original problem. While MUSCLE was unable to align this problem directly, using Crumble we were able to apply it to this problem.

#### Prune

To evaluate the performance of Prune on real world problems, we considered alignment problems from the Rfam seed alignment database with more than 200 sequences per RNA family [23]. This criteria gave twelve families: tRNA, 5S_rRNA, SRP_bact, MIR807, Cobalamin, PK-G12rRNA, SSU_rRNA_5, RNaseP_bact_a, tmRNA, SAM, U2, and U6. We tested the generated alignments against the hand curated "seed" alignment maintained by Rfam using the agreement score (Table 5). As for the simulated data in Table 3, we see non-monotonicity in agreement score: as Prune breaks the tree into smaller and smaller sub-trees, the agreement score starts to decrease but then begins to increase. Prune's performance on this data set is not as good as on simulated data. But it does significantly decrease run-time with only small to moderate loss of accuracy.

**Table 5:** Prune results for twelve alignment problems from the Rfam database.

		Time	**Agree**.
		
Pecan^1^		_*a*	_*a*
Prune w/Pecan	60%	_*a*	_*a*
	30%	14.6	0.651
	15%	5.35	0.649
	7%	2.57	0.643
FSA^2^		13.6	0.792
Prune w/FSA	60%	10.3	0.669
	30%	4.30	0.615
	15%	2.39	0.636
	7%	2.17	0.636

MUSCLE^3^		3.67	0.709
Prune w/MUSCLE	60%	3.03	0.704
	30%	1.23	0.649
	15%	1.03	0.672
	7%	1.42	0.659

MAFFT^4^		0.04	0.693

SATé^5^		93.9	0.753

^1 ^Pecan was run with default parameters.

^2 ^FSA was run with the --exonerate, --anchored, --softmasked, and --fast flags.

^3 ^MUSCLE was run with default parameters.

^4 ^MAFFT was run with --treein option.

^5 ^SATé was run with the -t option but limited to two iterations. We found that more iterations did almost nothing for accuracy.

^*a *^The majority of problems were unable to be aligned due to running out of memory.

The run-time and agreement score of Prune alignments of twelve RNA alignment problems from the Rfam database. The average time and agreement over all twelve problems are shown. Pecan, FSA, and MUSCLE were used as the underlying alignment method of Prune. MAFFT and SATé were also tested to provide comparison. We were unable to apply Pecan without using Prune because of memory issues. Using Prune, we were able to use Pecan to solve these alignment problems. Prune achieved a very large speedup with little loss of accuracy. Other alignment methods achieved a large speedup but more accuracy was lost.

### Future Development

The results presented here are for RNA and DNA alignment problems. While Crumble and Prune can be used to align protein sequences, their performance on this problem remains to be measured.

Crumble and Prune achieve their performance gains by leveraging smaller problem sizes and taking advantage of parallelization. The relative contributions of these two factors to overall performance has yet to be explored for the various alignment algorithms and problem sizes.

The next major step in the development of these methods is to adapt them to align regions that have undergone chromosomal translocations and inversions. The methods presented here can be used after programs such as Mercator [35] have unscrambled the region. A more integrated approach could better take advantage of the performance gains possible.

We also aim to adapt these methods to work on various cloud computing platforms such as Amazon's Elastic Compute Cloud.

## Conclusions

We have presented two general methods, Crumble and Prune, for improving the running time of alignment programs. The methods work by breaking large alignment problems into smaller sub-problems, solving those sub-problems, and reassembling them to form the full alignment. The sub-problems are formed so that many of them can be solved in parallel. This allows modern computer cluster systems to be leveraged to solve large alignment problems.

We have tested Crumble and Prune on a very large set of simulated alignment problems. The test dataset includes both long (~1 megabase of sequence) and deep (1000 species) alignment problems. Crumble and Prune were able to dramatically improve the run-time of Pecan, FSA, and MUSCLE on long and deep alignment problems with very little loss in alignment accuracy. In some cases, Prune was also able to improve the accuracy of FSA and MUSCLE while, at the same time, providing a boost in performance. We also tested Crumble and Prune on a set of biological data. While these data sets are relatively small with respect to the scale of problems that Crumble and Prune are designed to handle, they show that our methods do provide a significant performance improvement with only moderate loss of accuracy.

With Crumble and Prune we were able to apply Pecan, FSA, and MUSCLE to much longer and deeper problems than could be solved by running those programs without Prune because of memory or time constraints. This extends the applicability of these methods to larger alignment problems.

We believe that these methods will enable the application of more sophisticated and statistically motivated alignment algorithms toward large, real world alignment problems.

## Availability

• Project name: Crumble, Prune, Job-tree

• Project home page: http://hgwdev.cse.ucsc.edu/~krish/crumble_prune/. Datasets used to test the alignments are available at: http://hgwdev.cse.ucsc.edu/~krish/test_alignments/.

• Operating System(s): Linux 2.6.18

• Programming language: C++, Python

• Other requirements: Boost C++ Libraries 1.46

• License: GNU GPL

Crumble, Prune, and Job-tree are licensed under the GPL and and available for download at: http://hgwdev.cse.ucsc.edu/~krish/crumble_prune/. The datasets used to test the alignments are available at: http://hgwdev.cse.ucsc.edu/~krish/test_alignments/.

## Authors' contributions

KMR wrote the implementation, participated in software design, performed the benchmarking, and drafted the manuscript. BP participated in software design, assisted with the implementation, and helped draft the manuscript. DH participated in the design and coordination. All authors read and approved the final manuscript.
